# Editorial: Active Vision and Perception in Human-Robot Collaboration

**DOI:** 10.3389/fnbot.2022.848065

**Published:** 2022-02-08

**Authors:** Dimitri Ognibene, Tom Foulsham, Letizia Marchegiani, Giovanni Maria Farinella

**Affiliations:** ^1^Department of Psychology, Università degli Studi di Milano-Bicocca, Milan, Italy; ^2^School of Computer Science and Electronic Engineering, University of Essex, Colchester, United Kingdom; ^3^Department of Psychology, University of Essex, Colchester, United Kingdom; ^4^Department of Electronic Systems, Aalborg University, Aalborg, Denmark; ^5^Department of Mathematics and Computer Science, University of Catania, Catania, Italy

**Keywords:** active vision, social perception, intention prediction, egocentric vision, natural human-robot interaction, human-robot collaboration

## 1. Applying Principles of Active Vision and Perception to Robotics

Finding the underlying design principles which allow humans to adaptively find and select relevant information (Tistarelli and Sandini, 1993; Findlay and Gilchrist, 2003; Krause and Guestrin, 2007; Friston et al., 2015; Ognibene and Baldassare, 2015; Bajcsy et al., 2017; Jayaraman and Grauman, 2018; Ballard and Zhang, 2021) is important for Robotics and related fields (Shimoda et al., 2021; Straub and Rothkopf, 2021). Active inference, which has recently become influential in computational neuroscience, is a normative framework proposing one such principle: action, perception, and learning are the result of minimization of variational free energy, a form of prediction error. Active vision and visual attention must involve balancing long and short-term predictability and have been the focus of several previous modeling efforts (Friston et al., 2012, 2015; Mirza et al., 2016). Parr et al. review several probabilistic models which are needed for different aspects of biological active vision. They propose a mapping between the involved operations and particular brain structures.

Van de Maele et al. use deep neural networks to implement an active inference model of active perception, working in a rendered 3D environment similar to a robotics setting. Their network learns the necessary generative model of visual data and when tested shows interesting exploratory behavior. However, they also highlight the many computational challenges that must be solved before such a system can be tested on real robots with tasks to perform and humans to interact with.

Due to this high computational complexity, in practice, robotics scenarios often substitute optimal active perception strategies with flexible architectures that allow the development of behaviors for different tasks. Martin et al. introduce a scalable framework for service robots that efficiently encodes precompiled perceptual needs in a distributed knowledge graph.

## 2. The Challenge of Social Interactions

Social interactions involve non trivial tasks, such as intention prediction (Sebanz and Knoblich, 2009; Ognibene and Demiris, 2013; Donnarumma et al., 2017a), activity recognition (Ansuini et al., 2015; Lee et al., 2015; Sanzari et al., 2019) or even simple gesture recognition (e.g., pointing at a target), which may require perceptual policies that are difficult to precompile. This is because they are contingent on previous observations, hierarchically organized (Proietti et al., 2021), and must extend over time, space and scene elements which may not be always visible (Ognibene et al., 2013). While some active recognition systems and normative models for action and social interactions have already been proposed (Ognibene and Demiris, 2013; Lee et al., 2015; Donnarumma et al., 2017a; Ognibene et al., 2019b), it is not completely clear what strategy humans adopt in such tasks, not least because of the heterogeneity of the stimuli. Salatiello et al. introduce a validated generative model of social interactions that can generate highly-controlled stimuli useful for conducting behavioral and neuroimaging studies, but also for the development and validation of computational models.

An alternative approach is to simplify the challenges posed by social interactions by adopting a strict signaling and interaction protocol. Papanagiotou et al. investigate a collaborative human-robot industrial assembly task powered by an egocentric perspective (where the camera shares the user's viewpoint) and where the system must recognize gestures.

## 3. Transposing Active Perception Strategies From Ecological Interactions to Human Robot Collaboration

However, a better understanding of active vision and eye movements during social interaction may lead to more natural interfaces. Of course one of the most important ways in which humans interact is through speech. While there is a long tradition of studying the relationship between speech and gaze for behavior analysis, there is much less investigation with modern computational tools. Aydin et al. take a step in this direction by providing a multimodal analysis and predictors of eye contact data. This analysis reveals patterns in real conversation - such as the tendency for speakers to look away from their partner (Ho et al., 2015). In a similar context, D'Amelio and Boccignone introduce a novel computational model replicating visual attention behaviors while observing groups speaking on video. The model is based on a foraging framework where individuals must seek out socially relevant information. Testing these models with social robots would enable principled and natural conversational interaction but also determine if humans would find it effective (Palinko et al., 2016).

In ecological conditions where participants act in the world, gaze dynamics can also be highly informative about intentions (Land, 2006; Tatler et al., 2011; Borji and Itti, 2014; Ballard and Zhang, 2021). Wang et al. verify this hypothesis in a manipulation and assembly task to create a gaze-based intentions predictor covering multiple levels of the action hierarchy (action primitives, actions, activities) and study the factors that affect response time and generalization over different layouts.

## 4. Specificity of Gaze Behaviors During Human Robot Interaction

When Fuchs and Belardinelli studied the impact of a similar ecological approach to perform an actual teleoperation task, they found that gaze dynamics are still informative and usable. Interestingly, the patterns observed might partially differ from those in natural eye-hand coordination, probably due to limited confidence in robot behavior. While they expect that users would eventually learn an effective strategy, they suggest that more adaptive and personalized models of the effect of robot behavior on user gaze would further improve the interaction.

Eldardeer et al. developed a biologically inspired multimodal framework for emergent synchronization and joint attention in human-humanoid-robot interaction. The resulting interaction was robust and close to natural, but the robot showed slower audio localization due to ambient noise. While specific audio processing methods (Marchegiani and Newman, 2018; Tse et al., 2019) may ameliorate this issue, it highlights the importance of a detailed understanding of the temporal aspects of active perception and attention resulting from the interplay between exploration and communication demands in the human robot collaboration context (Donnarumma et al., 2017b; Ognibene et al., 2019a).

As these works show, human attentional and active perception strategies while interacting with a robot are interesting in their own right (Rich et al., 2010; Moon et al., 2014; Admoni and Scassellati, 2017). In ecological conditions, behavior with a robot will be different from performing the task alone (free manipulation), using a tool and even from collaborating with a human partner. At the same time, aspects of each situation will be reproduced, since robots can be perceived as body extensions, tools or companions. Following Fuchs and Belardinelli, we should expect the balance between these factors to shift after experience with a particular design of robot (Sailer et al., 2005).

To understand how humans and robots interact (and how they can interact better), a sensible place to start is by comparing this to how humans interact with each other. Czeszumski et al. report differences in the way that participants respond to errors in a collaborative task, depending on whether they are interacting with a robot or another person. Moreover, there were differences in neural activity in the two situations. This is an example of how researchers can begin to understand communication between humans and robots, while also highlighting potential brain based interfaces which could improve this communication.

## 5. Conclusions

Ultimately this collection of articles highlights the potential benefits of deepening our understanding of active perception and the resulting egocentric behavior in the context of human robot collaboration. Some of the challenges for future research are to:

1. Scale normative frameworks to deal with realistic tasks and environments (see Van de Maele et al. and Ognibene and Demiris, 2013; Lee et al., 2015; Donnarumma et al., 2017a; Ognibene et al., 2019b).
2. Enable scalable frameworks to deal with the uncertain, multimodal, distributed, and dynamic nature of social interactions (see Eldardeer et al., Martin et al., and Ognibene et al., 2013; Schillaci et al., 2013).
3. Deepen the integration of user state, e.g., beliefs (Bianco and Ognibene, 2019; Perez-Osorio et al., 2021), inference, into predictive models.
4. Improve egocentric perception (Grauman et al., 2021) and interfaces (see Papanagiotou et al.) to build advanced wearable assistant and to balance usability and robustness.
5. Understand and exploit the peculiarities of Human AI interactions (see Fuchs and Belardinelli, Czeszumski et al., and Paletta et al., 2019).
6. Provide new benchmarks and datasets (see Salatiello et al. and Ammirato et al., 2017; Damen et al., 2018; Calafiore et al., 2021).

## Author Contributions

All authors listed have made a substantial, direct, and intellectual contribution to the work and approved it for publication.

## Funding

DO and TF were supported by the European Union's Horizon 2020 research and innovation programme under grant agreement (No. 824153 POTION).

## Conflict of Interest

The authors declare that the research was conducted in the absence of any commercial or financial relationships that could be construed as a potential conflict of interest.

## Publisher's Note

All claims expressed in this article are solely those of the authors and do not necessarily represent those of their affiliated organizations, or those of the publisher, the editors and the reviewers. Any product that may be evaluated in this article, or claim that may be made by its manufacturer, is not guaranteed or endorsed by the publisher.
